# Battery Life of Pulse Generators in Spinal Cord Stimulation: Analysis and Comparison Between Surgical and Percutaneous Leads in Energy Efficiency

**DOI:** 10.3390/jcm14186646

**Published:** 2025-09-21

**Authors:** Marta Antonia Gómez González, Nicolás Cordero Tous, Carlos Sánchez Corral, Beatriz Lechuga Carrasco, Manuel Alejandro Sánchez García, Rafael Gálvez Mateos, Gonzalo Olivares Granados

**Affiliations:** 1 Functional Neurosurgery Unit, Department of Neurosurgery, Hospital Universitario Virgen de las Nieves, Juan Pablo II Avenue, S/N, 4th Floor, 18013 Granada, Spain; martaa.gomez.sspa@juntadeandalucia.es (M.A.G.G.); carlos.sanchez.corral.sspa@juntadeandalucia.es (C.S.C.); beatriz.lechuga.carrasco.sspa@juntadeandalucia.es (B.L.C.); gonzalo.olivares.sspa@juntadeandalucia.es (G.O.G.); 2 Pain Unit, Department of Anesthesiology, Reanimation and Pain Management, Hospital Universitario Virgen de las Nieves, Juan Pablo II Avenue, S/N, 2nd Floor, 18013 Granada, Spain; manuel.sanchez@ugr.es (M.A.S.G.); rmergalz@ugr.es (R.G.M.); 3 Anatomy Department, University of Granada, Doctor Jesus Candel Fabregas Avenue, 11, 18016 Granada, Spain

**Keywords:** spinal cord stimulation, pulse generator, failed back syndrome, complex regional pain syndrome, chronic pain

## Abstract

**Background:** Spinal cord stimulation (SCS) is an established therapy for chronic neuropathic pain. Although rechargeable and non-rechargeable pulse generators (PGs) are widely used, their real-world battery life and the influence of lead type on energy efficiency remain underexplored. **Objective:** To evaluate PG battery longevity and compare the performance of surgical versus percutaneous leads in terms of energy efficiency. **Methods:** We conducted a retrospective study of 283 PGs implanted at Hospital Virgen de las Nieves (Granada, Spain) from 1996 to 2023. Data on patient demographics, pain etiology, lead type and placement, stimulation modality, and PG status were extracted. A competing risks analysis was used to assess PG shutdown and early explantation over time. **Results:** Of the PGs analyzed, 43.5% were non-rechargeable and 56.5% rechargeable. Rechargeable PGs showed significantly longer battery life (mean: 82.7 vs. 38.9 months, *p* < 0.05), with a lower probability of shutdown at 50, 100, and 150 months. No significant differences in battery longevity were observed regarding lead location, stimulation type, or pain etiology. A trend toward longer battery life was observed with percutaneous leads, although not statistically significant. **Conclusions:** Rechargeable PGs demonstrated superior longevity compared to non-rechargeable models and should be considered the preferred option in most cases. While both surgical and percutaneous leads are effective, percutaneous systems may offer improved battery efficiency. Further prospective studies are warranted to confirm these findings and assess cost-effectiveness.

## 1. Introduction

Spinal cord stimulation (SCS) is a well-established therapy for chronic neuropathic pain [1,2], recognized as a safe procedure with low complication rates reported in the literature [1,3,4,5]. Multiple studies have demonstrated its superiority over conservative management alone [5,6]. From an economic perspective, SCS has been shown to be cost-effective over extended time horizons in patients with failed back surgery syndrome (FBSS) and complex regional pain syndrome (CRPS), with reported savings exceeding £100,000 per quality-adjusted life year gained [7].

When first introduced in 1967 [8], only surgical leads were available, requiring general anesthesia and laminectomy for implantation. Later, in 1975, percutaneous leads were developed to avoid general anesthesia, allowing implantation under local anesthesia in an outpatient setting [8]. This enables patients to remain awake during the procedure, providing real-time feedback to the surgeon to ensure stimulation coverage of the painful area.

Clinical comparisons have shown similar outcomes in pain relief between the two systems, although surgical leads tend to be associated with a higher rate of implant-related complications [1,9,10,11,12,13]. These leads are connected to a pulse generator (PG), which may be either rechargeable or non-rechargeable. Non-rechargeable PGs are less costly upfront but have a variable lifespan depending on programming parameters and stimulation intensity. In contrast, rechargeable PGs, while more expensive, offer longer battery life and could reduce the need for replacement surgeries over time. This, however, is based on technical specifications from manufacturers, and has yet to be proved in clinical settings.

Rechargeable PGs also support a wider range of stimulation modalities. Tonic stimulation, the earliest developed modality, typically delivers low-frequency (40–60 Hz) stimulation at amplitudes sufficient to elicit paresthesia in the painful region, based on the gate control theory of pain [14,15]. Over the past decade, paresthesia-free stimulation modalities—such as burst stimulation, high-frequency stimulation (ranging from 1 kHz to 10 kHz), and proprietary waveform patterns (e.g., DTM, FAST)—have been introduced [15]. Although the underlying mechanisms of action for these newer modalities remain incompletely understood [15], they provide effective pain relief without inducing paresthesia. However, these approaches generally have higher energy demands, necessitating more frequent recharging, typically on a weekly or even more frequent basis [14].

To date, no studies have specifically evaluated the battery lifespan of PGs. Moreover, there is a lack of comparative data assessing the efficiency of surgical versus percutaneous lead systems in terms of PG battery longevity. Therefore, in this study, we aim to analyze the battery lifespan of both rechargeable and non-rechargeable PGs and investigate whether lead type (surgical vs. percutaneous) has an impact on battery duration.

## 2. Materials and Methods

Following regional ethical approval (Study NC-D-01, Ethics Committee Reference: SICEIA-2020-000438), we conducted a retrospective review of all patients who underwent spinal cord stimulator (SCS) implantation at Hospital Virgen de las Nieves (Granada, Spain) between November 1996 and December 2023. As part of this research, we previously described the implantation protocol employed at our center in a prior publication [16].

In our center, a multidisciplinary committee composed of anesthesiologist, neurologist, neurosurgeons, neurophysiologist and rehabilitations specialists meets monthly, to asses patients with chronic pain who may benefit for interventional therapies such as SCS. The inclusion criteria for a patient to be eligible for SCS is shown in Box 1.

Box 1 **Inclusion criteria for SCS. Adapted from Gómez-González et al. [16].**

Chronic pain in a specific area of the body (patients experiencing diffuse pain were not accepted)

Failure of conservative treatments, including rehabilitation and local infiltration

A favorable psychological evaluation confirming that the patient can wear a spinal cord stimulation system

No contraindications to surgery or allergies to system components

No history or current substance use, especially opioids

### 2.1. Implantation Procedure

The primary distinction between surgical and percutaneous leads lies in their implantation technique. Surgical leads are placed under general anesthesia, typically at the upper level of T8 for patients with failed back surgery syndrome (FBSS) and at the C2 level for those with complex regional pain syndrome (CRPS) in their upper limbs. However, intraoperative confirmation of optimal lead placement over the painful region is not possible, as neuromonitoring is not performed during these procedures. In contrast, percutaneous leads are implanted under local anesthesia, following the same anatomical landmarks. A paresthesia-guided stimulation technique is employed during implantation to confirm adequate pain coverage in real time, allowing intraoperative lead repositioning to optimize therapeutic outcomes.

After the implantation of one type of leads, a trial phase is performed, in which the leads are connected to an external stimulator. The efficacy of the system was then assessed over a 4-week period using the verbal numerical rating score. If the patient’s reported subjective perception of pain relief was at least 50%, the trial phase was successful, so a permanent PG was implanted. When a patient did not achieve the threshold, the system was removed completely.

**Figure 1:**
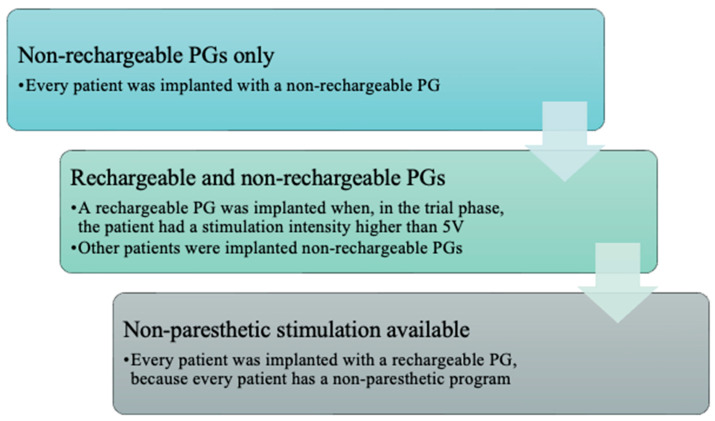
Election of PGs through the years.

In earlier cases, only non-rechargeable PGs were implanted, as rechargeable devices were not yet available at our center. As newer stimulation modalities became accessible, a series of implantation protocols were developed and approved by a multidisciplinary committee, including anesthesiologists, neurologists, neurosurgeons, neurophysiologists, and rehabilitation specialists. These protocol changes are summarized in Figure 1. At present, all patients receive rechargeable PGs since 2017, regardless of the type or intensity of stimulation used during the trial phase. This practice has been adopted because all patients are ultimately programmed with non-paresthetic stimulation programs, even if tonic stimulation is also included in their treatment settings.

### 2.2. Data Collection and Analysis

To assess PG battery longevity, we included all PGs implanted during the study period. No devices were excluded, as our aim was to evaluate battery lifespan in a real-world clinical setting. Each PG was analyzed individually. Data were extracted retrospectively from the electronic medical records of each patient. Prior to data collection, all patients were informed about the study protocol and provided written informed consent.

The following variables were collected at the time of implantation: patient demographics (age, sex), etiology of pain, lead type associated with the PG (surgical or percutaneous), lead location, stimulation modality, type of PG (rechargeable or non-rechargeable), PG status at the end of the study (active or explanted), and whether the PG experienced an early shutdown. A PG shutdown was defined as battery depletion necessitating replacement. An early shutdown was defined as the explantation of a functioning PG due to other causes, and is considered a competitive risk, as the PG is still working when explanted, so we cannot assess battery life correctly. Stimulation modality was categorized as tonic or non-paresthetic (including burst, and high-frequency stimulation from 1 to 10 kHz). In some cases, we did not find the specific PG that was implanted, only whether it was rechargeable or non-rechargeable. Thus, we could not conduct an economic analysis, as we usually implant systems from different brands.

To evaluate PG battery life, the implantation and explantation dates of each device were recorded, looking at surgical notes to know the exact date. If a PG was explanted, the reason for removal was documented, distinguishing between pocket problems, device-related infection, lack of pain relief, hardware malfunction, death of the patient or battery depletion. When the patient passed away, we considered the date of the death certificate as the explantation date, as we could no longer assess battery life.

Data analysis was performed using SPSS^®^ 20.0 (IBM; Armonk, NY, USA). A competing risks analysis was conducted to estimate the probability of PG shutdown and early shutdown over time, using reference time points of 50, 100, and 150 months. For rechargeable PGs with different stimulation modalities, time points were adjusted to 50 and 80 months due to shorter follow-up durations. This statistical model provided failure probabilities over time for both rechargeable and non-rechargeable PGs, as well as early shutdown probabilities during follow-up.

The competing risks analysis was also repeated across different lead types, stimulation modalities, and other variables to assess their potential impact on PG longevity. A *p*-value of <0.05 was considered statistically significant.

## 3. Results

Between November 1996 and December 2023, a total of 283 PGs were implanted at Hospital Virgen de las Nieves in Granada, Spain. Of these, 123 (43.46%) were non-rechargeable, while the remaining 160 (56.54%) were rechargeable. A total of 141 PGs (49.82%) were connected to surgical leads, whereas 142 (50.18%) were paired with percutaneous leads. Tonic stimulation was employed in 240 cases (84.81%), and non-paresthesia-inducing stimulation was used in 43 cases (15.19%). At the conclusion of the study, 134 PGs (47.35%) were no longer in use, with 56 (19.79%) experiencing early shutdown.

Analyzing the cause for early shutdown, we had 10 (17.86%) explantations due to pocket pain, 4 (7.14%) due to PG exposition, 8 (14.29%) due to infection, 5 (8.93%) due to hardware malfunction, 15 (26.79%) due to change in stimulation, 8 (14.29%) due to lack of pain relief, 5 (8.93%) due to death of the patient and 1 (1.77%) due to an epidural hematoma.

Non-rechargeable PGs had a mean battery life of 38.89 ± 24.83 months (mean ± standard deviation), with a maximum of 79 months. Rechargeable PGs demonstrated a mean battery life of 82.69 ± 45.88 months, reaching up to 173 months. When excluding devices that experienced early shutdown, the battery life of 96 non-rechargeable PGs increased to 59.60 ± 50.99 months, while 131 rechargeable PGs had a battery life of 50.99 ± 33.11 months (*p* = 0.08). By the end of the follow-up period, 24 non-rechargeable and 125 rechargeable PGs remained operational. A comparative analysis between non-rechargeable and rechargeable PGs is presented in Table 1.

**Figure 2:**
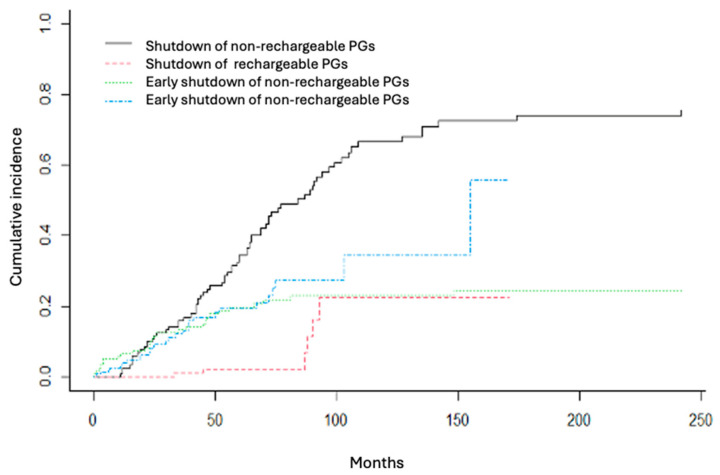
Competitive risk analysis to study battery life. In black, shutdown of non-rechargeable PG; in red, shutdown of rechargeable PG; in green, early shutdown of non-rechargeable PG; in blue, early shutdown of rechargeable PG.

A competing risk analysis was conducted to exclude all PGs that were explanted for reasons unrelated to battery depletion. The probability of shutdown, when comparing the battery life of rechargeable versus non-rechargeable PGs, was found to be statistically significant throughout the entire follow-up period, up to 150 months (*p* < 0.05). In contrast, the probability of early shutdown remained comparable between both groups over the same period (*p* = 0.45). Detailed data on shutdown and early shutdown probabilities are presented in Table 2 and Table 3, respectively. The competing risk analysis is illustrated in Figure 2.

To compare PG battery life between surgical and percutaneous paddles, two separate analyses were performed. Firstly, we performed and analysis including all PGs, and later, another limited to rechargeable PGs was performed, as all PGs implanted with percutaneous paddles were rechargeable. This was made to minimize technology bias. The overall analysis indicated a trend toward shorter battery life in PGs associated with surgical paddles compared to those paired with percutaneous leads (probability of shutdown: 0.18 vs. 0.10 at month 50, 0.49 vs. 0.41 at month 100, and 0.53 vs. 0.60 at month 125; *p* = 0.08). The competing risk analysis showed no significant difference between the groups in terms of early shutdown (*p* = 0.55).

**Figure 3:**
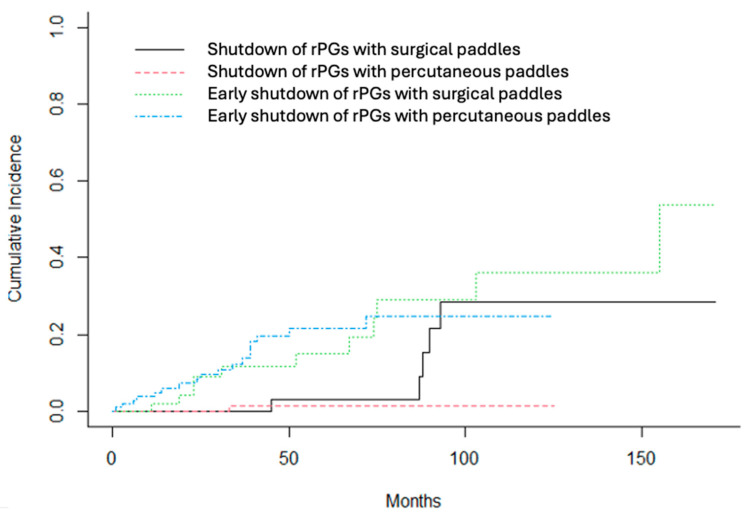
Competitive risk analysis to study battery life depending on type of paddle, using only rechargeable PGs. In black, shutdown of PGs with surgical paddles; in red, shutdown of PGs with percutaneous paddles; in green, early shutdown of PGs with surgical paddles; in blue, early shutdown of PGs with percutaneous paddles.

When the comparison between surgical and percutaneous paddles was restricted to rechargeable PGs, only six devices had failed by the end of follow-up. The probability of shutdown showed a temporal trend, with earlier failure in PGs connected to surgical paddles compared to those with percutaneous leads (0.02 vs. 0.01 at month 50, 0.28 vs. 0.01 at month 100, and 0.28 vs. 0.01 at month 125); however, this difference did not reach statistical significance (*p* = 0.22). Similarly, the competing risk analysis revealed no significant difference between groups for early shutdown (*p* = 0.79). A detailed comparison of PG battery life by paddle type is presented in Table 4 and Table 5, and illustrated in Figure 3.

**Figure 4:**
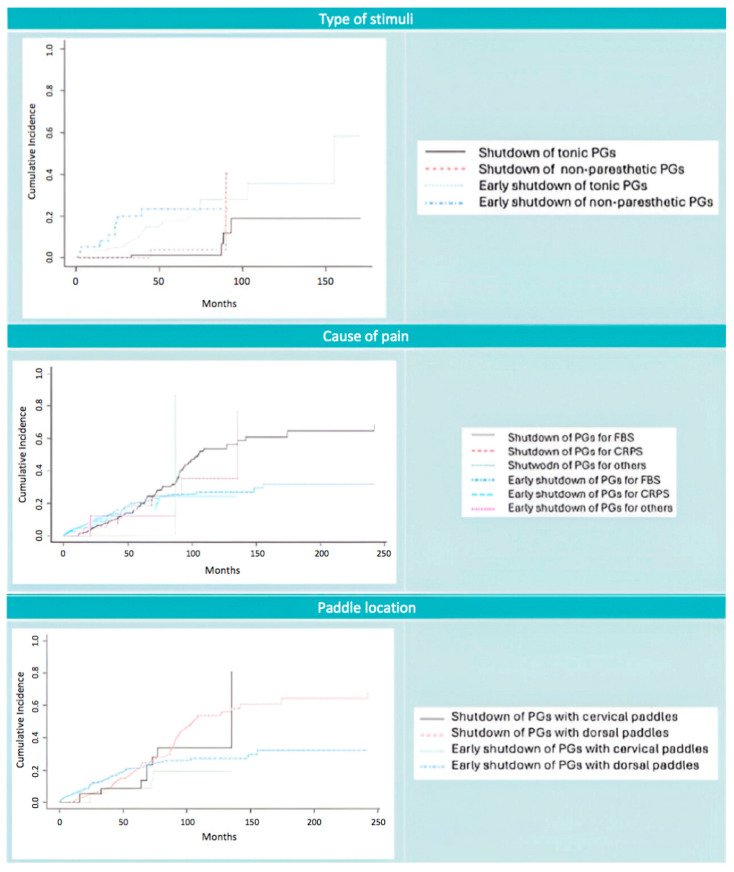
Competitive risk analysis to study battery life depending on different variables. On the left side, graphical representation. On the right side, color interpretation.

With respect to stimulation type, only rechargeable PGs were analyzed to assess both overall shutdown and early shutdown. This was made to minimize technological bias. In this context, no statistically significant differences were observed in the probability of shutdown over time (0.01 vs. 0.03 at month 50; 0.12 vs. 0.40 at month 92; *p* = 0.21). Similarly, no significant differences were found regarding early shutdown in this subgroup analysis. Furthermore, battery life did not significantly differ based on lead localization or pain etiology, regardless of whether PGs were rechargeable or non-rechargeable. These results are summarized in Table 6 and Table 7, and illustrated in Figure 4.

## 4. Discussion

Spinal cord stimulation (SCS) is a well-established treatment for chronic neuropathic pain, including failed back surgery syndrome (FBSS) and complex regional pain syndrome (CRPS) [1,2]. The system consists of one or two leads placed in the epidural space, connected to a pulse generator (PG), which may be either non-rechargeable or rechargeable. Battery longevity varies depending on the manufacturer; currently, both types of PGs are advertised as having a lifespan of up to 10 years [17,18]. However, these figures are based on manufacturer-reported data. Independent studies have reported mean battery life for non-rechargeable PGs ranging from 28 to 50 months [19,20], although newer models may demonstrate improved longevity.

To date, comparisons of battery life have primarily focused on economic evaluations. A 2010 cost-effectiveness analysis [21] assumed a battery life of 9 years for rechargeable PGs and less than 4 years for non-rechargeable PGs and was limited to patients with CRPS. Similarly, a 2020 study [22] reported a longer median battery life for rechargeable PGs compared to non-rechargeable models (7.20 years vs. 3.68 years, respectively). Another cost-consequence analysis published in 2008 [23] estimated the average battery life of non-rechargeable PGs to be approximately 49 months (4.1 years), based on previous literature, while rechargeable PGs were projected to last more than 10 years.

In our study, we measured that non-rechargeable PGs had a battery life of 59.60 ± 50.99 months (4.96 years), whereas there were 131 rechargeable PGs with a battery life of 50.99 ± 33.11 months (4.24 years). However, 125 of the 131 rechargeable PGs were still working by the end of the study, so we believe this time is shortened artificially. In addition, when we performed the competitive risk analysis, we found that the probability of shutdown was higher for non-rechargeable PGs at 50, 100 and 150 months (*p* < 0.05), thus we believe that median battery life is also higher for rechargeable PGs in our study. With this in mind, rechargeable PGs should be implanted in most patients, as they have outperformed non-rechargeable PGs during all follow up, including the first months after implantation, at least according to our data.

In a 2021 study, Luecke et al. [24] analyzed data from a German database to compare non-rechargeable and rechargeable PGs. They found no significant differences in patient age or pain etiology between the two groups. However, the explantation rate was notably higher in patients with non-rechargeable PGs (34 vs. 15). In contrast, our study examined the probability of experiencing a competing risk event and found no statistically significant difference between non-rechargeable and rechargeable PGs. Based on these findings, we propose that both device types have a similar likelihood of explantation.

We also investigated battery longevity among rechargeable PGs based on the type of stimulation. No statistically significant difference was observed in time to shutdown (0.01 vs. 0.03 at month 50; 0.12 vs. 0.40 at month 90; *p* = 0.21), nor in the competing risk analysis. In this context, a 2022 study [25] developed a computational model of the dorsal horn network to simulate various stimulation patterns and their effects. The authors concluded that 50 Hz stimulation resulted in greater inhibition of spontaneously firing neurons, which may enhance the efficacy of SCS and potentially extend PG battery life.

When comparing rechargeable PG battery life by lead type, we observed a higher probability of shutdown in rechargeable PGs connected to surgical leads compared to percutaneous ones (0.02 vs. 0.01 at month 50; 0.28 vs. 0.01 at months 100 and 125; *p* = 0.22), although this difference did not reach statistical significance. This may be attributed to the relatively small number of rechargeable PGs paired with surgical leads, as most were implanted with percutaneous leads. Additionally, many of the surgical leads in our cohort were implanted earlier, raising the possibility of technological bias or increased fibrosis surrounding the lead. We believe that using only rechargeable PGs minimizes the technological bias, as all patients had been implanted with this type of PGs from 2017 to 2023, but further studies comparing both newer PGs and newer electrodes should be made to confirm if this trend could have a clinical impact.

A modeling study suggests that the most effective stimulation for FBS is a transverse tripolar arrangement, that requires a surgical lead or three parallel percutaneous leads [26]. In this study, the surgical lead recruited a slightly larger and deeper dorsal column nerves than the percutaneous leads, but it also required higher energy for stimulation than the percutaneous lead. However, in a clinical setting, percutaneous leads are more likely to be surrounded by epidural fat, thus potentially requiring more energy than surgical leads. In addition, surgical leads push the dura ventrally, reducing the distance between the surgical lead and the neural target, so the authors conclude than, in a clinical setting, using staggered quadripolar lateral anodes configured on a 3-column surgical lead can provide an improved performance than the same configuration with percutaneous leads in treating low-back pain complaints [27]. We must acknowledge that most surgical paddles in our study were implanted before 2010, so we cannot assess the effect of fibrosis in our study, and we do not know if this changes energy efficiency for surgical leads.

Costandi et al. [22] also assessed that the median cost per day was similar for both IPGs, $13.90 and $13.81 for non-rechargeable and rechargeable, respectively. The median cost for SCS systems was higher for the rechargeable group ($60.70) compared with the non-rechargeable group ($31.38). In this line, Tito et al. [28] compared battery life between three different non-rechargeable PGs and the cost of implantation. They concluded that one non-rechargeable PG (Medtronic Vanta PC) had a longer battery life compared to other non-rechargeable PG (9.27 years and 5.1 years) and implanting them could save €2712–4227 compared to using other non-rechargeable PG. In our study we did not focus on the economic comparison between rechargeable and non-rechargeable PG, so further studies should be made.

Even if our study does not include an economic analysis, we present the first study to our knowledge that analyses battery life of PGs in a real-life setting. As we did not have concrete information about which model of PGs were implanted in earlier patients, we could not compare how PG longevity in a real-life setting correlates with the information provided by the different manufacturers. However, it is a starting point that should be further explored, as longevity provided by manufacturers usually is an estimation based on the capacity of the battery. In addition, we found that rechargeable PGs had a greater battery life than non-rechargeable PGs, since most rechargeable PGs were still working by the end of the study. Using rechargeable PFs could, potentially, minimize the number of surgeries to change the PG, thus minimizing the costs associated with the surgical procedure and complications. This should be explored in other studies.

The first limitation that our study presents is, therefore, the lack of economic analysis between rechargeable and non-rechargeable PG, as it is an important factor to consider when deciding the type of PG to be implanted. Another important limitation is that technology, especially regarding PGs, has been evolving since the first PG implanted in 1996 in our hospital. In this regard, we are comparing non-rechargeable and rechargeable PGs that have different battery capacity; therefore, they could have different battery life in a clinical setting. With this in mind, we aim to provide a base to know battery life in a real clinical setting, but further studies should be made to assess difference between newer non-rechargeable and rechargeable PGs. Also, more studies should be made from an economic perspective, comparing non-rechargeable and rechargeable PGs and correlating battery life and economic costs.

## 5. Conclusions

Spinal cord stimulation (SCS) is a cost-effective therapy for managing chronic neuropathic pain. Rechargeable pulse generators (PGs) demonstrate statistically significant longer longevity, even within a 24-month period, and therefore should be considered the preferred option in most cases. Both surgical and percutaneous leads are electrically efficient; however, rechargeable PGs paired with percutaneous leads may exhibit longer battery life.

In our analysis, no statistically significant differences in battery longevity were observed based on stimulation type, lead location, or pain etiology. Further studies are warranted to evaluate potential differences in battery performance between PGs connected to surgical versus percutaneous leads, as surgical leads may offer greater energy efficiency under certain conditions.

## Figures and Tables

**Table 1:** Demographic analysis with follow up time in months.

	Non-Rechargeable PGs (*n* = 123)	Rechargeable PGs (*n* = 160)
Cause of pain		
FBS	92 (74.80%)	107 (66.88%)
CPRS	30 (24.39%)	43 (26.88%)
Others	1 (0.81%)	10 (6.25%)
Type of lead		
Surgical	86 (69.92%)	55 (34.38%)
Percutaneous	37 (30.08%	105 (65.62%)
Lead location		
Cervical	13 (10.57%)	32 (20.00%)
Dorsal	110 (89.43%)	128 (80.00%)
Type of stimulation		
Tonic	118 (95.93%)	122 (76.25%)
Non-paresthetic	5 (4.07%)	38 (23.75%)
Shutdown at the end		
Yes	24 (19.51%)	125 (78.12%)
No	99 (80.49%)	35 (21.88%)
Early shutdown		
Yes	27 (21.95%)	29 (18.12%)
No	96 (78.05%)	131 (81.88%)
Time of life		
Mean ± standard deviation	38.89 ± 24.83	82.69 ± 45.88
Minimum–maximum	0–79	9–173

**Table 2:** Probability of shutdown of non-rechargeable and rechargeable PG at 50, 100 and 150 months, and comparison between them.

Time	Non-Rechargeable PG (*n* = 96)	Rechargeable PG (*n* = 131)	*p*
50 months	0.25 (25%)	0.01 (1%)	<0.05
100 months	0.60 (60%)	0.22 (22%)	
150 months	0.72 (72%)	0.22 (22%)	

**Table 3:** Probability of early shutdown of non-rechargeable and rechargeable PG at 50, 100 and 150 months, and comparison between them.

Time	Non-Rechargeable PG (*n* = 27)	Rechargeable PG (*n* = 29)	*p*
50 months	0.17 (17%)	0.16 (16%)	0.45
100 months	0.22 (22%)	0.27 (17%)	
150 months	0.24 (24%)	0.34 (34%)	

**Table 4:** Probability of shutdown of PGs depending on the type of paddle.

Time	All PG		Rechargeable PG	
	Surgical Paddle (*n* = 108)	Percutaneous Paddle (*n* = 119)	Surgical Paddle (*n* = 5)	Percutaneous Paddle (*n* = 1)
50 months	0.18 (18%)	0.10 (10%)	0.02 (2%)	0.01 (1%)
100 months	0.49 (49%)	0.41 (41%)	0.28 (28%)	0.01 (1%)
125 months	0.53 (53%)	0.60 (60%)	0.28 (28%)	0.01 (1%)
*p* value	0.08		0.22	

**Table 5:** Probability of early shutdown of PGs depending on the type of paddle.

Time	All PG		Rechargeable PG	
	Surgical Paddle (*n* = 33)	Percutaneous Paddle (*n* = 23)	Surgical Paddle (*n* = 11)	Percutaneous Paddle (*n* = 18)
50 months	0.17 (17%)	0.17 (17%)	0.17 (17%)	0.20 (20%)
100 months	0.27 (27%)	0.27 (27%)	0.29 (29%)	0.25 (25%)
125 months	0.28 (0.28%)	0.21 (21%)	0.36 (36%)	0.25 (25%)
*p* value	0.55		0.79	

**Table 6:** Probability of shutdown and early shutdown (competitive risk) of rechargeable PGs at 50 and 80 months.

Variable	*p* Value			50 Months	80 Months
Type of stimulation	Shutdown: 0.21 Early shutdown: 0.74	Tonic (*n* = 240)	Shutdown (*n* = 227)	0.01 (1%)	0.12 (12%)
			Early shutdown (*n* = 13)	0.14 (14%)	0.27 (27%)
		Non-paresthetic (*n* = 43)	Shutdown (*n* = 35)	0.03 (3%)	0.4 (4%)
			Early shutdown (*n* = 8)	0.23 (23%)	0.23 (23%)

**Table 7:** Probability of shutdown and early shutdown of non-rechargeable and rechargeable PGs at 50 and 100 months depending on paddle placement and cause of pain.

Variable	*p* Value			50 Months	100 Months	150 Months
Paddle placement	Shutdown: 0.19 Early shutdown: 0.74	Cervical (*n* = 45)	Shutdown (*n* = 40)	0.08 (8%)	0.19 (19%)	0.19 (19%)
			Early shutdown (*n* = 5)	0.08 (8%)	0.33 (33%)	0.33 (33%)
		Dorsal (*n* = 238)	Shutdown (*n* = 187)	0.18 (18%)	0.25 (25%)	0.27 (27%)
			Early shutdown (*n* = 51)	0.14 (14%)	0.47 (47%)	0.56 (56%)
Cause of pain	Shutdown: 0.88 Early shutdown: 0.90	FBS (*n* = 199)	Shutdown (*n* = 155)	0.17 (17%)	0.24 (24%)	
			Early shutdown (*n* = 44)	0.13 (13%)	0.3 (3%)	
		CRPS (*n* = 73)	Shutdown (*n* = 62)	0.15 (15%)	0.23 (23%)	
			Early shutdown (*n* = 11)	0.15 (15%)	0.25 (25%)	
		Others (*n* = 11)	Shutdown (*n* = 10)	0.12 (12%)	0.23 (23%)	
			Early shutdown (*n* = 1)	0.00 (0%)	0.00 (0%)	

## Data Availability

The datasets presented in this article are not readily available because of ethical reasons.

## Abbreviations

The following abbreviations are used in this manuscript:

SCS	Spinal cord stimulation
FBS	Failed back syndrome
CRPS	Complex regional pain syndrome
PG	Pulse generator
